# *De Novo* Genome Sequencing and Assembly of Rhodosporidium toruloides Strain Δdao1e

**DOI:** 10.1128/mra.00600-22

**Published:** 2023-01-18

**Authors:** Xiao Guo, Shuobo Shi

**Affiliations:** a Beijing Advanced Innovation Center for Soft Matter Science and Engineering, College of Life Science and Technology, Beijing University of Chemical Engineering, Beijing, China; University of California, Riverside

## Abstract

Rhodosporidium toruloides is an important industrial strain with high oil production. Here, we report the *de novo* sequencing of *R. toruloides* strain Δdao1e, which was derived from *R. toruloides* strain ATCC 10657, by combining sequencing results from the Illumina and PacBio Sequel platforms.

## ANNOUNCEMENT

Rhodosporidium toruloides, a basidiomycetous yeast that belongs to the order *Sporidiobolales* and subphylum *Pucciniomycotina* (1), was originally isolated in Dalian, China, in 1922 (2). Currently, several strains of *R. toruloides* have been isolated from various environments and sequenced (https://www.ncbi.nlm.nih.gov/genome/browse#!/eukaryotes/13280/). *R. toruloides* strain Δdao1e (haploid; A2 mating type) is a *KU70* gene-deficient and *DAO1*-null mutant derived from a *R. toruloides* strain ATCC 10657 background; it displays a greatly improved phenotype in inducible gene expression and homologous recombination (3). It is an important strain in metabolic engineering and synthetic biology.

A single colony was cultivated in 10 mL of yeast-peptone-dextrose (YPD) medium (1.0% yeast extract, 2.0% peptone, 2.0% glucose) for 24 h at 30°C. Genomic DNA was extracted using a yeast genomic DNA extraction kit (DP307; Tiangen, China) and sent for sequencing (BGI Genomics Co., Ltd., China). The libraries for the extracted DNA were constructed using the MGIEasy stLFR library prep kit (MGI Technology Co., Ltd., China).

Paired-end (2 × 300-bp) sequencing was performed using the Illumina and PacBio Sequel platforms. In order to obtain more accurate and reliable results in the subsequent bioinformatics analysis, the raw reads were quality controlled and treated using fastp v0.20.0 to filter low-quality data (4). Falcon (parameters, -v -dal8 -t32 -h60 -e.96 -l500 -s100 -H3000) (5) was used to perform the *de novo* assembly. Finally, SSPACE_Basic_v2.0 (with default parameters) (6) was chosen for scaffold construction.

The genome of *R. toruloides* strain Δdao1e is 21,178,773 bp long and contains 42,546,318 paired-end reads with an average of 150 bp for a genome coverage of 263×. The genome was assembled into 25 contigs and 19 scaffolds with an *N*_50_ value of 1,460,187 bp and a GC content of 61.49%, generating 41,365 gaps. In total, 8,126 genes were predicted combining GeneWise (applying the sequencing results of *R. toruloides* strain NP11 [GenBank assembly accession number GCA_000320785.2] for alignment of evidence) (7) and GeneMark-ES (8) software, with 51,323 exons and 43,197 introns. Functional annotation was performed using BLAST (9) to search 23 databases, including the NCBI nonredundant (NR) database, Gene Ontology (GO) database, and Kyoto Encyclopedia of Genes and Genomes (KEGG). Since there was more than one alignment result per sequence, the highest quality alignment result was chosen for the gene annotation to ensure the biological meaning; eventually, 8,043 protein-encoding genes were annotated. Furthermore, 29 rRNAs, 220 tRNAs, and 30 small RNAs (sRNAs) were predicted using RNAmmer software (10), tRNAscan (11), and Infernal (12), respectively.

In a previous study, *R. toruloides* strain ATCC 10657 was assembled into 234 contigs and 137 scaffolds, with a scaffold *N*_50_ of 1,053,226 bp and a contig *N*_50_ of 378,481 bp (GenBank accession number LNKU00000000.1). In this research, we performed whole-genome sequencing of the mutant strain *R. toruloides* Δdao1e with improved accuracy and continuity and assembled it into 25 contigs and 19 scaffolds, with a scaffold *N*_50_ of 1,460,187 bp and a contig *N*_50_ of 1,368,862 bp. Furthermore, indels exist in this assembly compared to the parental strain through sequence alignment with BLASTN. Specifically, there are a 1,882-bp deletion and an 81-bp insertion in the *KU70* gene encoding ATP-dependent DNA helicase 2 located between positions 1993277 and 1994522 in scaffold 2 of our genome and between positions 1286607 and 1289653 in scaffold 5 of the parental genome. There is a 912-bp deletion and an 87-bp insertion in the *DAO1* gene encoding d-amino acid oxidase located between positions 722288 and 724976 in scaffold 6 of our genome and between positions 723590 and 727103 in scaffold 3 of the parental genome.

### Data availability.

This whole-genome shotgun project has been deposited at DDBJ/ENA/GenBank under the accession number JAMRMT000000000.1. The version described in this paper is version JAMRMT010000000. The BioSample accession number is SAMN28102265, and the BioProject accession number is PRJNA835422.
